# Blood and skin carotenoid levels are inversely associated with the prevalence of periodontal diseases in populations with normal occlusion: a cross-sectional analysis from the Iwaki health promotion project

**DOI:** 10.1186/s12937-026-01285-y

**Published:** 2026-01-28

**Authors:** Toshitaka Yamauchi, Naoko Waki, Shigenori Suzuki, Kenji Fujimoto, Tatsuya Mikami, Koichi Murashita, Shigeyuki Nakaji, Ken Itoh, Yoshinori Tamada, Yoshihiro Tamura, Wataru Kobayashi

**Affiliations:** 1 Department of Vegetable Life Science, Graduate School of Medicine, Hirosaki University, Hirosaki, Japan; 2 Department of Medical Data Intelligence, Research Center for Health-Medical Data Science, Graduate School of Medicine, Hirosaki University, Hirosaki, Japan; 3 Diet & Well-being Research Institute, KAGOME CO., LTD., Nasushiobara, Japan; 4 Department of Electronics and Information Technology, Graduate School of Science and Technology, Hirosaki University, Hirosaki, Japan; 5 Department of Preemptive Medicine, Innovation Center for Health Promotion, Hirosaki University Graduate School of Medicine, Hirosaki, Japan; 6 Research Institute of Health Innovation, Hirosaki University, Hirosaki, Japan; 7 Department of Stress Response Science, Biomedical Research Center, Hirosaki University Graduate School of Medicine, Hirosaki, Japan; 8 Department of Oral and Maxillofacial Surgery, Hirosaki University Graduate School of Medicine, Hirosaki, Japan

**Keywords:** Oral health, Periodontal diseases, Antioxidants, Skin carotenoid, Normal occlusion, Bayesian network analysis

## Abstract

**Background:**

Oral health has a significant effect on longevity and quality of life. Periodontal diseases and dental caries are the primary causes of tooth loss, with the former being more prevalent than the latter. Antioxidants are associated with periodontal diseases. However, comprehensive analyses of this association are limited in literature, especially when focusing on circulating antioxidants. This study analyzed the association between antioxidant levels in the blood and skin along with periodontal diseases in populations with normal occlusion.

**Methods:**

This cross-sectional study was conducted as part of the Iwaki Health Promotion Project in 2022. Overall, 456 individuals aged ≥ 20 years with healthy occlusion were included. Multivariate logistic regression analyses were performed, with periodontal disease defined as the primary outcome variable, circulating antioxidant levels as the exposure, and the following covariates: age, sex, smoking and drinking status, sugar intake, educational background, and oral care habits. In addition, the potential causal direction between antioxidant levels and periodontal diseases were explored using Bayesian network analysis.

**Results:**

Individuals with the fifth quintile of blood and skin carotenoid levels had a lower odds ratio (OR) for periodontal diseases compared with those with the first quintile (blood: adjusted OR = 0.32 [95% confidence interval (CI), 0.16–0.64], skin: adjusted OR = 0.45 [95% CI, 0.23–0.88]). In particular, blood lutein and lycopene levels, but not vitamin A, C, and E levels, were significantly associated with periodontal diseases. Bayesian network analysis suggested that carotenoid levels may be a potential causal factor for periodontal diseases, and this association may be mediated by salivary immunoglobulin A and oral dysbiosis.

**Conclusions:**

Blood and skin carotenoid levels are associated with the prevalence of periodontal diseases.

**Supplementary Information:**

The online version contains supplementary material available at 10.1186/s12937-026-01285-y.

## Background

Oral health has a significant effect on longevity and quality of life [1, 2]. Oral disorders, metabolic syndrome, locomotive syndrome, and dementia are major contributors to the decline in healthy life expectancy among older individuals [1]. Notably, individuals with fewer remaining teeth have exhibited a significantly higher risk of cardiovascular and respiratory diseases, cancer, long-term care dependency, and mortality [3–6]. The proposed mechanisms include indirect mental and social along with direct biological involvement [6–11]. Regarding direct involvement, local inflammation caused by periodontitis triggers systemic inflammation, resulting in a high risk of cardiovascular diseases [7]. Additionally, invasion of oral pathogens into the digestive tract induces inflammatory bowel diseases [8]. Regarding indirect involvement, negative emotion about inability to eat well or enjoy meals triggers social withdrawal, engendering a high dementia risk through the reductions in the number of social contacts and physical activity levels [6, 9–11].

Periodontal diseases and dental caries are the primary causes of tooth loss. Approximately 40% of tooth extractions in Japan are due to periodontal diseases, and approximately 30% are caused by dental caries [12]. In particular, periodontal diseases are prevalent in two of three individuals aged ≥ 30 years, and many of them are asymptomatic [12]. The onset and progression of periodontal diseases involve oral bacteria, especially red complex bacteria, such as *Porphyromonas gingivalis*, *Tannerella forsythia*, and *Treponema denticola* [13, 14]. Bacterial toxins, such as gingipains, induce gingival inflammation and contribute to the deepening of the periodontal pockets [15]. The deepening of the periodontal pockets promotes the accumulation of dental plaque, triggering a vicious cycle that exacerbates the condition and ultimately results in tooth loss. The representative risk factors for periodontal disease include poor oral hygiene, smoking, and obesity (or hyperglycemia) [16–21]. Proper oral hygiene practices are effective in reducing periodontal plaque and inhibiting the growth of red complex bacteria [16, 17]. Smoking increases the risk of periodontal diseases by promoting salivary acidification, inducing hypoxia, and impairing host immune regulation [18, 19]. Hyperglycemia damages the capillary walls surrounding the gingiva and increases the levels of inflammatory mediators, resulting in a high risk of periodontal diseases [20, 21].

Oxidative stress and antioxidants are associated with periodontal diseases [22]. Notably, high intakes of antioxidants, such as β-carotene and vitamin C, causes a low risk of the onset of periodontal diseases [23–25]. However, to the best of our knowledge, these studies have relied on food questionnaires, and no comprehensive studies have investigated the association between circulating antioxidants and periodontal disease. Although intakes of lycopene and vitamin E improve periodontal diseases in patients [26], studies involving healthy individuals are limited.

In this study, we comprehensively analyzed the association between antioxidant levels in the blood and skin along with periodontal diseases in populations with normal occlusion using data from the Iwaki Health Promotion Project (IHPP) in 2022 [27]. In addition, we explored the potential causal direction between antioxidant levels and periodontal diseases using a Bayesian network analysis.

## Materials and Methods

### Study design

This study included participants of the IHPP in 2022 [27]. This is a health examination targeting men and women aged ≥ 20 years living in northern Japan. Recruitment was conducted by inviting residents of the target area through our website and other local announcements. Demographic and lifestyle information was collected using questionnaires. Antioxidant levels were measured in blood samples. Oral microbiome data were obtained from tongue-coated samples for Bayesian network analyses.

Participants were excluded based on the following criteria: missing relevant data, history of gastrointestinal cancer, or unhealthy occlusion (Eichner classifications B1–C3). The Eichner classification is a system for assessing occlusal support based on the presence or absence of functional tooth contacts in the premolar and molar regions. Individuals are classified into groups A1–A3, B1–B4, and C1–C3 based on the number and distribution of occlusal supporting zones, thereby enabling an evaluation of masticatory function and prosthodontic requirements.

- Group A1–A3: All four occlusal supporting zones are present.
- Group B1–B4: One to three occlusal supporting zones, or posterior support is absent but anterior contact remains.
- Group C1–C3: No occlusal contacts in either the anterior or posterior regions.

Participants with unhealthy occlusions were excluded to minimize the possibility of reverse causation. Antioxidants, particularly carotenoids, are abundant in vegetables, which are relatively difficult to consume. Deterioration of occlusion may lead to a decrease in vegetable intake, and such reverse causation is undesirable in the regression analysis.

### Clinical evaluation of health status

The following markers were measured: body mass index (BMI), probing depth, Eichner classification, presence of calculus and gingival bleeding, number of dental caries, salivary immunoglobulin A (IgA) levels, and salivary flow rate. Dental markers were evaluated by four examiners who underwent calibration training prior to data collection to standardize assessment procedures. Probing depth was assessed using the four-site method (mesiobuccal, distobuccal, mesiolingual/palatal, and distolingual/palatal sites). For each tooth, the deepest pocket depth among the four sites was recorded. The participants were classified as having periodontal diseases if the maximum depth was ≥ 4 mm. Saliva was collected by allowing participants to rest quietly while a sterile sponge absorbed saliva in the oral cavity for 1 min, without any mechanical or gustatory stimulation. Salivary IgA concentrations were quantified using enzyme-linked immunosorbent assay (Healthcare Systems Co. Ltd., Nagoya, Japan).

### Questionnaires

The questionnaire included items regarding age, sex, educational background, smoking and drinking status, medical history, dietary habits, oral care habits (number of brushing, use of floss and interdental brush, and dental visit status in the last one year), and oral health indices (Oral Health Impact Profile-14: OHIP14). Dietary habits, including sugar intake and vegetable intake, were assessed using the larger 172-item Food Frequency Questionnaire (FFQ) developed for the Japan Public Health Center-based Prospective Study for the Next Generation and previously validated for use in Japanese populations [28]. Food and nutrient intake were calculated using the designated computer software (FFQ NEXT, Kenpakusha, Tokyo, Japan) based on the Standard Tables of Food Composition in Japan 2020 (eighth revised edition).

### Quantification of antioxidants (carotenoids and vitamins A, C, and E) in the blood

We measured lutein, zeaxanthin, β-cryptoxanthin, α-carotene, β-carotene, lycopene, and vitamin A, C, and E levels in blood as antioxidants. To quantify antioxidant levels, we followed previous studies [29, 30]. Briefly, all antioxidants, excluding vitamin C, were extracted from serum samples using a mixture of *n*-hexane and dichloromethane (4:1, v/v), followed by filtration. The solvent was then evaporated, and the residue was reconstituted in a mixed solution of methanol/acetonitrile/dichloromethane (7:7:2, v/v/v) for analysis by high-performance liquid chromatography (Prominence LC-30AD/Nexera X2 SPPD-M30A; Shimadzu Co., Kyoto, Japan). Vitamin C was extracted from plasma samples and quantified using a commercial kit (R01K02; Shima Laboratories Co., Ltd., Tokyo, Japan).

The total blood carotenoid concentration was defined as the sum of the concentrations of lutein, zeaxanthin, β-cryptoxanthin, α-carotene, β-carotene, and lycopene.

### Quantification of skin carotenoid

To quantify skin carotenoid levels, we followed the methods of a previous study [29]. Briefly, skin carotenoid levels were measured using Vegecheck^®^ (KAGOME CO., LTD., Aichi, Japan), which used a multiple spatially resolved reflection spectroscopy [31] sensor (Biozoom Services GmbH, Kassel, Germany), by placing the palm over the sensor. The results are expressed as skin carotenoid levels, ranging from 0.1 to 12.0.

### Microbiota analysis

To obtain oral microbiome data, we followed the steps of a previous study [32]. Briefly, tongue coating samples were collected before breakfast, and tooth brushing was performed on the day of the health checkup. The collected samples were stored at 4 °C in tubes containing a mixed solution (4 M guanidine thiocyanate, 100 mM Tris(hydroxymethyl) aminomethane hydrochloride [pH 8.0], 40 mM ethylenediaminetetraacetic acid, and 0.001% bromothymol blue) until analysis. DNA was extracted from the bead-processed samples using an automated nucleic acid extraction system GENE PREP STAR PI-480 (Kurabo Industries, Osaka, Japan) with a reagent kit NR-201 (Kurabo Industries). The V3–V4 region of the 16 S rRNA gene was amplified using a universal primer set, according to a previous report [33]. Sequencing was performed using the Illumina MiSeq system, and quality control, trimming, merging, and chimera detection were performed using DADA2 [34]. Bacterial taxonomy was assigned using the Ribosomal Database Project version 16 [35].

### Confounder selection

To determine confounders for the analysis, we followed a previous study [36]. Four criteria were proposed for the selection of confounders. The first is the backdoor criterion, which considers the variables that block backdoor paths as confounders. The second is the pretreatment criterion, which considers variables that occur prior to both exposure and outcome as confounders. The third criterion is the common cause criterion, which considers variables that cause both exposure and outcome as confounders. The fourth criterion is the modified disjunctive cause criterion, which considers variables that cause either exposure or outcome as confounders. This eliminates bias due to unmeasured or unknown confounders.

We assumed a causal diagram shown in Additional file 1 for this study and selected confounders based on the four criteria. Consequently, five variables (sex, age, smoking status, drinking status, and sugar intake) were selected based on the common cause criterion. This set of variables was defined as Model (1) In addition, educational background was selected as a confounder based on backdoor criterion, and was defined as Model (2) Finally, oral care habits were selected as confounders based on pretreatment and modified disjunctive cause criteria, and was defined as Model 3.

None of these criteria identify BMI or diabetes as confounders because these factors are mediators, which can introduce bias and potentially lead to under- or over-estimation of the effect size. Nevertheless, because high BMI and diabetes are well-known risk factors for periodontal disease, we additionally examined a model adjusted for BMI (Model 4).

### Statistical analyses

Multivariate logistic regression analysis was performed to analyze the association between antioxidant levels and periodontal diseases.

The E-value was calculated for sensitivity analysis. Statistical significance was set at *p* < 0.05. All analyses were performed using the R software (version 4.4.2).

### Bayesian network analysis

To explore the potential causal direction between antioxidant levels and periodontal diseases, we performed Bayesian network analysis. Bayesian network analysis has been successfully used to suggest potential causal associations among variables based on conditional dependency [37–40]. The INGOR software [41, 42] was used for the analysis. INGOR has the advantage of handling a large number of variables by employing a greedy hill-climbing algorithm and the bootstrap method on supercomputers. It suggests the network structure based on the BNRC score [41], i.e., a statistical criterion to evaluate the likelihood of the network structure based on Bayesian inference. In this study, we used 47 variables, including demographic information, lifestyle factors, dietary habits, and the oral microbiome (Additional file 2). Among the oral microbiomes, only the bacterial genera that comprise the red complex, namely *Porphyromonas*, *Tannerella*, and *Treponema*, and representative oral bacterial genera, namely *Neisseria*, *Prevotella*, and *Streptococcus*, were used to exclude an excessive number of variables. Age, sex, and educational background were designated as root nodes without parent nodes. Out of the 10,000-times bootstrap inferences, only the edges that appeared in > 5% of the iterations were included in the final network. The directions of the edges were illustrated based on the following criteria: if the direction of an edge was consistent in > 75% of the bootstrap iterations, it was represented by a single directed arrow; otherwise, a bidirectional arrow was used. The importance of each edge (gain) was defined as the change in the BNRC score caused by the presence or absence of the edge.

## Results

### Enrolled study participants

**Fig. 1:**
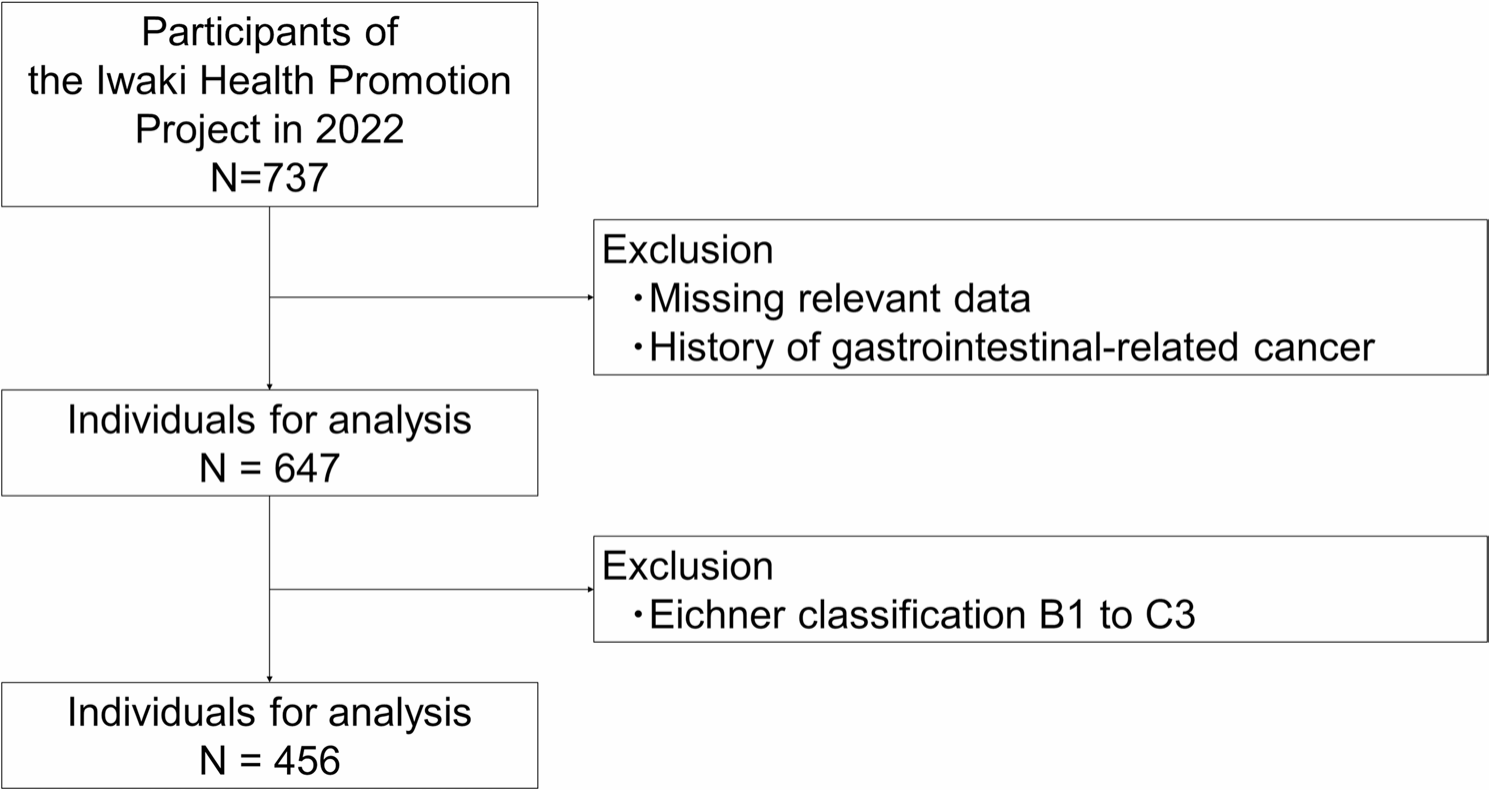
Flowchart of participant selection

The analysis included 456 of 737 IHPP participants in 2022 who did not meet any of the exclusion criteria (Fig. 1). The basic characteristics are listed in Table 1. The proportion of males was 39.5%, and the mean age was 48.0 years. The prevalence of periodontal disease was 50.9% (56.7% in males and 47.1% in females), which is similar to that reported in previous studies [12].

The prevalence of periodontal diseases differed according to known risk factors, such as age, smoking status, and BMI (Additional file 3).

**Table 1:** Characteristics of participants

		level/Unit	Overall	Male	Female
	*n*		456	180	276
Demographic information	Age	Years	48.0 ± 13.3	48.2 ± 12.9	47.9 ± 13.5
	Educational background	Junior High School	2.4 (11)	2.8 (5)	2.2 (6)
		High School	57.0 (260)	60.6 (109)	54.7 (151)
		Technical School	26.5 (121)	18.3 (33)	31.9 (88)
		College	14.0 (64)	18.3 (33)	11.2 (31)
Lifestyle	Smoking status	Never	54.8 (250)	30.0 (54)	71.0 (196)
		Current	16.2 (74)	28.3 (51)	8.3 (23)
		Past	28.9 (132)	41.7 (75)	20.7 (57)
	Drinking status	Never	44.5 (203)	26.7 (48)	56.2 (155)
		Current	50.9 (232)	69.4 (125)	38.8 (107)
		Past	4.6 (21)	3.9 (7)	5.1 (14)
	Sugar intake	g/day	0.19 ± 1.12	0.14 ± 0.75	0.22 ± 1.30
	Vegetable intake	g/day	215.4 ± 152.9	160.6 ± 117.1	251.1 ± 162.8
Oral care habits	Brushing	Number	2.3 ± 0.8	2.0 ± 0.8	2.5 ± 0.8
	Floss and interdental brush	Yes	52.6 (246)	40.6 (73)	60.5 (167)
	Dental Clinic (Last one year)	Yes	36.4 (166)	46.1 (83)	30.1 (83)
Circulating carotenoid	Carotenoid (Total)	µg/mL	1.37 ± 0.66	1.15 ± 0.52	1.52 ± 0.70
	Lutein	µg/mL	0.31 ± 0.15	0.28 ± 0.14	0.32 ± 0.15
	Zeaxanthin	µg/mL	0.08 ± 0.03	0.07 ± 0.03	0.08 ± 0.03
	β-cryptoxanthin	µg/mL	0.11 ± 0.07	0.09 ± 0.07	0.13 ± 0.07
	α-carotene	µg/mL	0.18 ± 0.16	0.15 ± 0.16	0.20 ± 0.16
	β-carotene	µg/mL	0.41 ± 0.34	0.27 ± 0.23	0.50 ± 0.37
	Lycopene	µg/mL	0.29 ± 0.17	0.28 ± 0.16	0.30 ± 0.17
	Vitamin A	µg/mL	0.57 ± 0.16	0.67 ± 0.16	0.50 ± 0.13
	Vitamin E	µg/mL	14.1 ± 3.7	14.2 ± 3.7	14.0 ± 3.8
	Vitamin C	µg/mL	9.4 ± 3.1	8.4 ± 3.1	10.0 ± 2.9
	Skin carotenoid		5.2 ± 1.3	4.7 ± 1.0	5.5 ± 1.3
Health status	Body mass index	kg/m/m	22.9 ± 3.3	23.7 ± 2.9	22.4 ± 3.4
	Teeth	Number	27.7 ± 2.2	27.9 ± 2.3	27.6 ± 2.1
	Periodontal diseaase	Yes	50.9 (232)	56.7 (102)	47.1 (130)

Each numerical variable is expressed as mean ± standard deviation, and each categorical variable is expressed as a percentage (N). *p*-value was calculated by the Kruskal-Wallis test (numerical variables) and Fisher’s exact test (categorical variables)

### Association between blood antioxidants concentrations or skin carotenoid levels and periodontal diseases

**Fig. 2:**
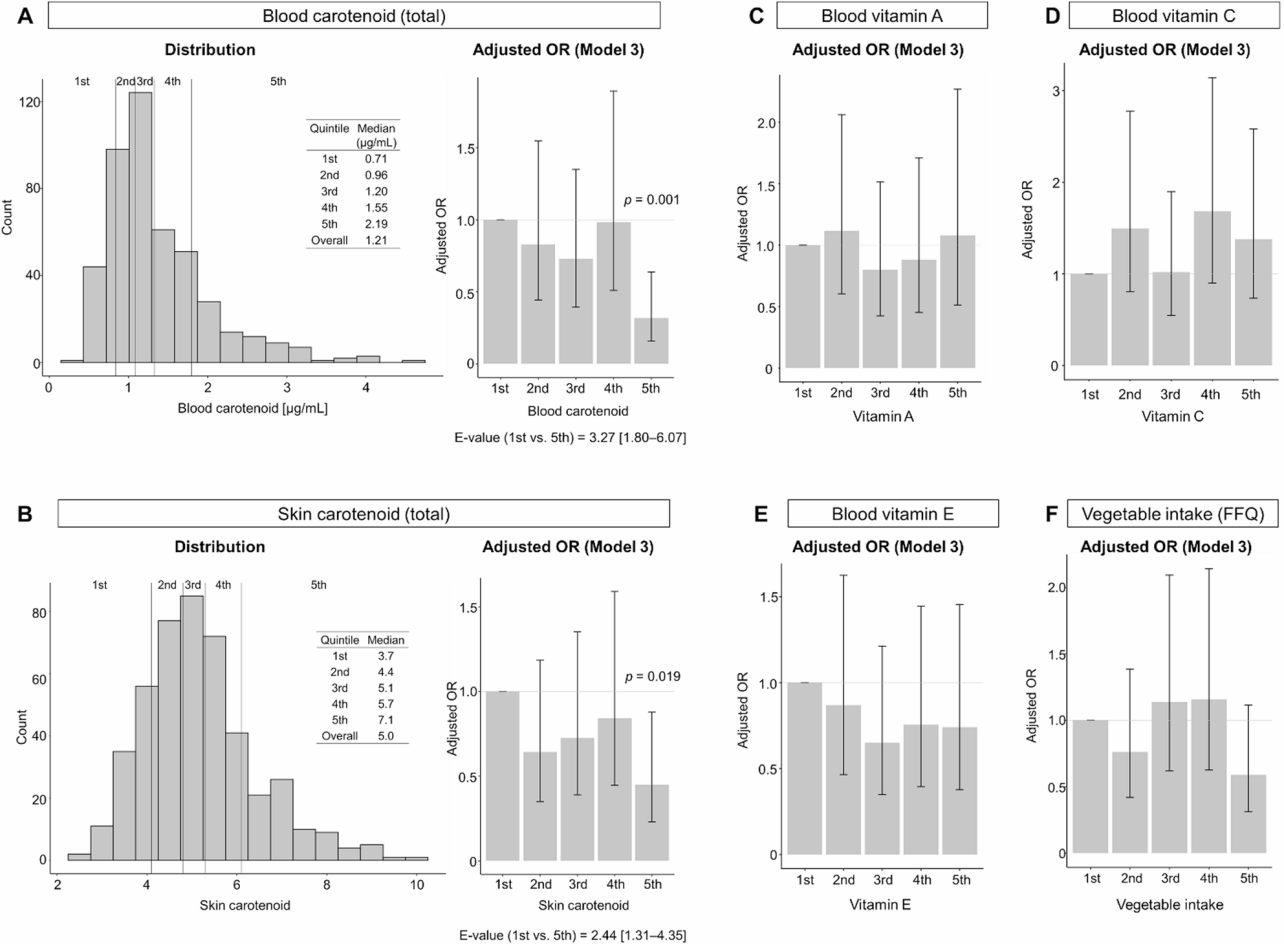
Histograms and odds ratios for periodontal diseases according to antioxidant levels (A–E) and vegetable intake (F) only odds ratios in model 3 were illustrated. The first quintile was used as a reference. Age, sex, smoking and drinking status, sugar intake, educational background, and oral care habits were considered confounders. Whiskers indicate 95% confidence intervals. OR, odds ratio

Multivariate logistic regression analysis revealed that individuals in the fifth quintile of blood and skin carotenoid levels had low adjusted odds ratios (ORs) for periodontal disease compared with those in the first quintile in all models (Fig. 2A, B, Additional file 4). Sensitivity analysis showed that the E-values for blood and skin carotenoid levels were 3.27 (95% confidence interval [CI], 1.80–6.07) and 2.44 (95% CI, 1.31–4.35), respectively. In contrast, vitamin A, C, and E levels were not significantly associated with periodontal diseases (Fig. 2C–E, Additional file 4).

Considering that carotenoids are abundant in vegetables, an additional analysis was performed to examine the association between vegetable intake and periodontal diseases. No significant associations were observed (Fig. 2F, Additional file 4).

**Fig. 3:**
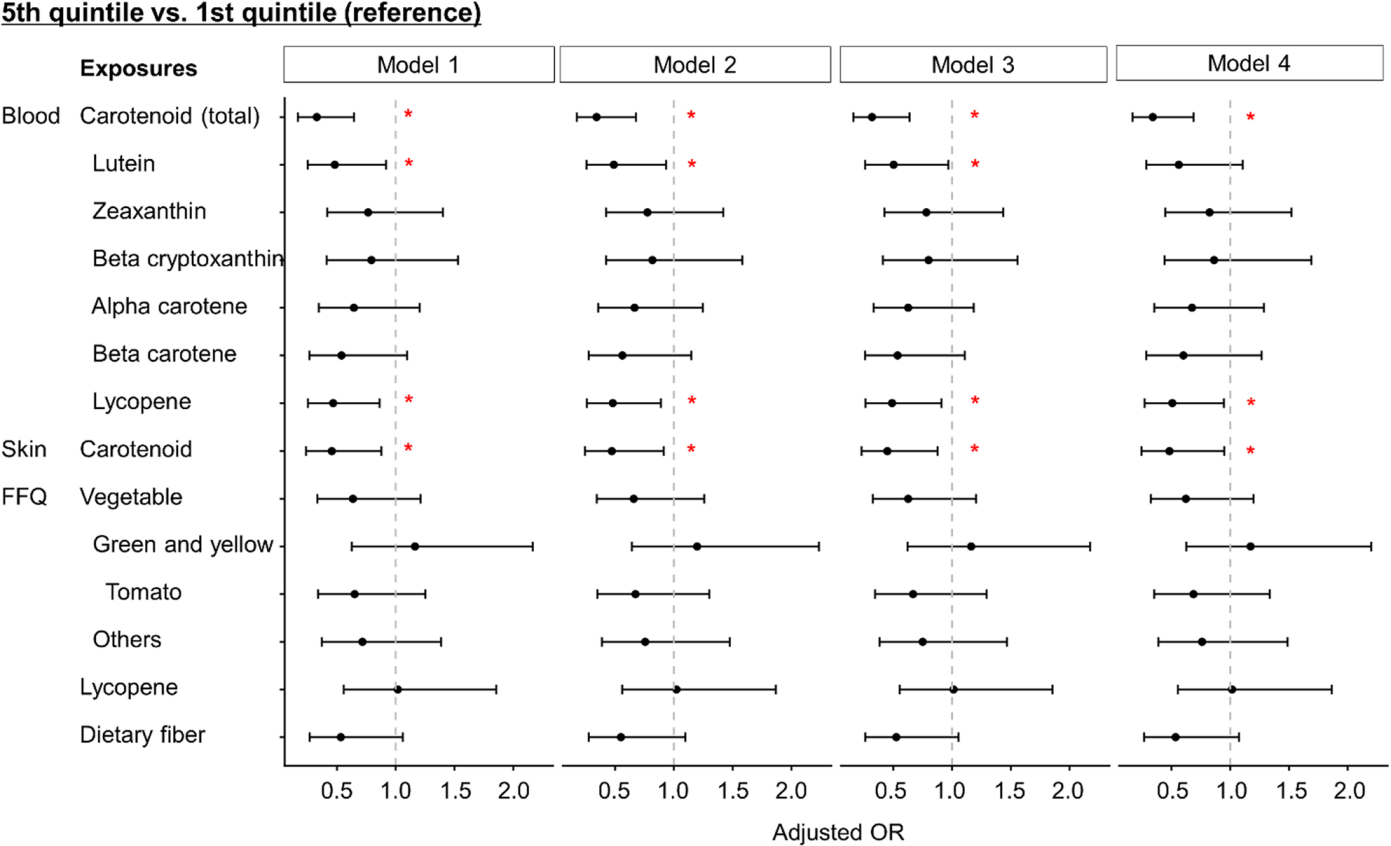
Odds ratios for periodontal diseases according to blood and skin carotenoid levels and dietary intake. Odds ratios for periodontal diseases in the fifth quintile relative to the first quintile of blood and skin carotenoid levels as well as dietary intake were illustrated. Dots and whiskers indicate odds ratios and 95% confidence intervals, respectively. The confounders used in each model were as follows: Model 1: sex, age, smoking and drinking status, and sugar intake; Model 2: Model 1 + educational background; Model 3: Model 2 + oral care habits; and Model 4: Model 3 + BMI. The results of blood carotenoid (total), skin carotenoid, and vegetable intake were restatements of the results shown in Fig. 2 and Additional files 4 and 5. OR, odds ratio; FFQ, Food Frequency Questionnaire. *p < 0.05

To further elucidate the associations, we analyzed the associations between periodontal diseases and blood concentration of each carotenoid (lutein, zeaxanthin, β-cryptoxanthin, α-carotene, β-carotene, and lycopene), as well as more detailed vegetable and nutrient intake assessed using a questionnaire. Hereafter, only the adjusted ORs comparing the fifth quintile to the first quintile are shown, as a significant association was detected in the preliminary analysis (Fig. 2). Blood lycopene levels were also associated with periodontal diseases in all models (Fig. 3, Additional file 5). In addition, blood lutein levels showed significant associations in Models 1–3. No significant associations were observed between periodontal diseases and intake of green and yellow vegetables, other vegetables, tomatoes, lycopene, or dietary fiber (as assessed using the questionnaire).

Subsequently, subgroup analyses on the association between carotenoid levels and periodontal diseases were performed according to sex, age, smoking status, and BMI. Additional File 6 presents the adjusted subgroup-specific odds ratios and the *p*-values for the interaction terms derived from the multivariate logistic regression. Using blood lycopene levels as the exposure variable, we found that the adjusted ORs were significantly lower in participants with a history of smoking and in older individuals.

### Results of Bayesian network analysis

**Fig. 4:**
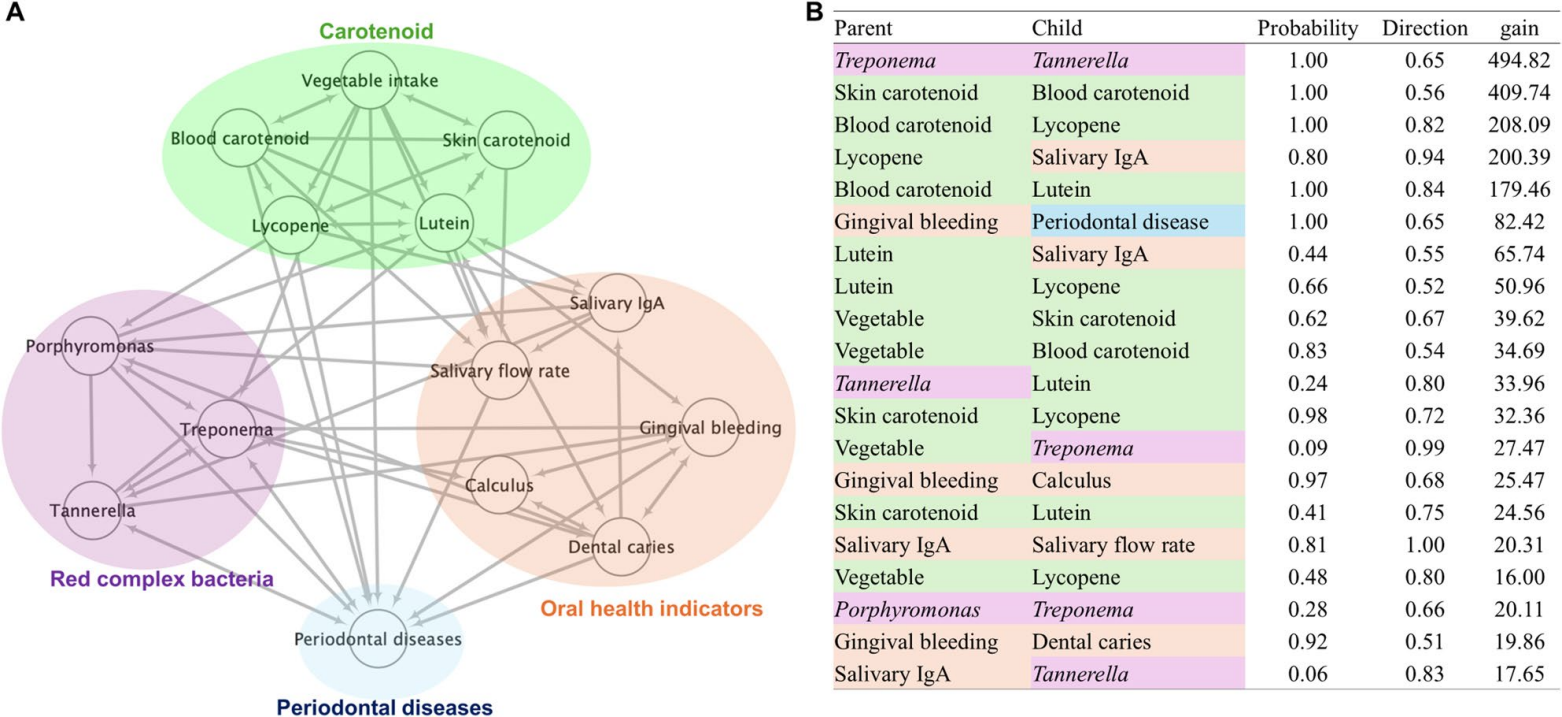
Potential causal directions and the top 20 most important edges. **A** Edges that appear with a proportion of ≥ 5% of 10,000 bootstraps were illustrated. The directions of the edges were illustrated based on the following criteria: if the direction of an edge was consistent in > 75% of cases, it was represented by a single directed arrow; otherwise, a bidirectional arrow was used. **B** Fill color of the cells correspond to categories of variables, green: carotenoid, purple: red complex bacteria, orange: oral health indicators, blue: periodontal diseases. The Direction column shows the proportion of these edges that were direct from the parent node to the child node. Gain indicates the improvement in goodness of fit attributable to the node

To statistically infer potential causal relationships and underlying mechanisms, we conducted Bayesian network analysis, revealing dense associations among the variables (Additional file 7). To facilitate interpretation, only the following nodes of interest were illustrated: carotenoid levels, periodontal disease, oral health indicators, and the bacterial genera that comprise the red complex (Fig. 4A). Nine variables were associated with periodontal disease: vegetable intake, blood carotenoid levels, lycopene, *Porphyromonas*, *Treponema*, *Tannerella*, salivary flow rate, dental caries, and gingival bleeding. In all these connections, periodontal disease was positioned downstream of these variables.

The top 20 most important edges (basesd on gain) is listed in Fig. 4B. Among the edges with the highest gain values, many were between variables within the same category. Nevertheless, edges connecting lycopene and lutein with salivary IgA also exhibited high gains. Although salivary IgA levels substantially varied among individuals, both lycopene and lutein levels were negatively associated with the median salivary IgA levels (Additional file 8). Considering that salivary IgA was associated with the red complex bacteria, the association between carotenoid levels and periodontal diseases may be potentially mediated by oral inflammation and dysbiosis.

## Discussion

Carotenoids contribute to the prevention of several diseases [43, 44]. The proposed mechanism involves the prevention of inflammation through antioxidant activity [23, 26], which is consistent with oral health. A previous study showed that under oxidative stress, chronic lipopolysaccharide (LPS) stimulation promoted macrophage-mediated inflammation, leading to the development of periodontal diseases [40]. However, under non-oxidative stress conditions, stimulation simultaneously induces anti-inflammatory cytokines, thereby preventing the onset of periodontal diseases. This suggests that the antioxidant activity of carotenoids may prevent inflammation and its onset, even in the presence of LPS stimulation by oral bacteria. Consistent with this suggestion, carotenoid levels were negatively associated with salivary IgA levels, a marker of oral inflammation [45], in the present study. Considering the edge from salivary IgA to red complex bacteria suggested by the Bayesian network analysis, carotenoids may be associated with periodontal diseases by attenuating oral dysbiosis via their anti-inflammatory activity.

However, it remains unclear whether this effect is specific to lutein and lycopene rather than to other carotenoids or vitamins. One possible hypothesis is the tissue-specific accumulation of carotenoids. Carotenoids predominantly found in the human body include lutein, zeaxanthin, β-cryptoxanthin, α-carotene, β-carotene, and lycopene. Certain carotenoids tend to accumulate selectively in specific tissues. For instance, lutein and zeaxanthin are highly concentrated in the retina and are strongly associated with ocular diseases [46]. Lycopene accumulates in the reproductive organs as well as contributes to improved fertility and the prevention of prostate cancer [47, 48]. Although there are no reports indicating that lutein and lycopene specifically accumulate in the gingiva, intervention with lutein and lycopene increases the concentrations of the corresponding carotenoids in the buccal mucosa [49, 50]. Thus, tissue-specific accumulation may lead to different effects among the carotenoids. Another hypothesis is the involvement of mechanisms other than antioxidant activity. Lycopene directly prevents the degradation of inhibitor of kappa B alpha, a repressor of the inflammation related-transcription factor nuclear factor kappa-light-chain-enhancer of activated B cells, exhibiting its anti-inflammatory activity [51]. Similarly, other unknown mechanisms of lutein and lycopene may lead to different effects among carotenoids.

Subgroup analysis revealed that blood lycopene levels were associated with periodontal diseases in older individuals, and those with a history of smoking. Thus, carotenoids may have a strong association on periodontal diseases in high-risk individuals.

Circulating carotenoid levels are associated with vegetable intake [52]. However, no association was found between vegetable intake and periodontal diseases in the present study. This finding highlights the importance of consuming carotenoid-rich foods and absorbing carotenoids effectively. However, considering that the adjusted OR for periodontal diseases according to vegetable intake tended to be < 1 (adjusted OR = 0.63 [95% CI, 0.33–1.20]; Model 3), this may be due to insufficient statistical power of questionnaire-based measures compared with biomarkers. This possibility is further supported by the fact that no association was observed between lycopene intake estimated by the questionnaire and periodontal diseases, whereas such an association was found with blood lycopene levels. In fact, several intervention and observational studies have reported an association between vegetable intake and periodontal diseases [53]. Moreover, the possibility is reinforced by the Bayesian network analysis results indicating that vegetable intake is linked to periodontal diseases. Taken together, the possibility that vegetable intake is associated with periodontal diseases cannot be excluded. Dietary fiber and carotenoids are abundant in vegetables. Our findings showed no association between dietary fiber intake and periodontal diseases. However, considering that the adjusted OR is 0.53 (95% CI, 0.26–1.05; Model 3) and that the analysis was based on questionnaire data, it is difficult to conclude that there is no association. A previous study reported the association [54], highlighting the need for further investigation. The proposed mechanisms include the regulation of immune function by short-chain fatty acids produced through the fermentation of dietary fiber by gut bacteria and promotion of salivary flow rate through the mastication of fibrous foods. Altogether, although we found no association between vegetable intake and periodontal diseases, a potential association through the effects of vegetables or dietary fiber cannot be eliminated.

We found an association between skin carotenoid levels and periodontal diseases. Skin carotenoid levels were measured noninvasively by placing the palm over the device for approximately 30 s. This method imposes less burden on the participants than dietary assessments using questionnaires. Considering that skin carotenoids are associated with metabolic syndrome-related biomarkers [29, 55], self-monitoring skin carotenoids serves as an effective tool to increase awareness of declining not only oral health but also systemic health. We found that individuals in the fifth quintile of skin carotenoid levels exhibited a lower adjusted OR for periodontal diseases. These individuals had a median skin carotenoid level of 7.1. A previous study also showed that the OR for metabolic syndrome progressively decreased as skin carotenoid levels approached approximately 7.0 [55], suggesting that using a skin carotenoid level of 7 as a target value in self-monitoring and health guidance is plausible. However, the proportion of individuals with skin carotenoid levels of ≥ 7 was less than 20% in this study, highlighting the need for interventions to increase carotenoid and vegetable consumption.

Owing to its cross-sectional design, it was difficult to identify a causal association in this study. Thus, we conducted Bayesian network analysis for potential causal discovery, a data-driven approach that suggests potential causal directions among variables by analyzing patterns of conditional dependency. Periodontal diseases were positioned downstream of carotenoids in the potential structure, suggesting that carotenoids could potentially have a protective effect against periodontal diseases. Variables other than carotenoids that are directly associated with periodontal diseases include red complex bacteria, salivary flow rate, and gingival bleeding. Additionally, although there were many edges extending from carotenoids to red complex bacteria and oral health indicators, the edges in opposite directions were limited. These results are in line with those of previous studies [14, 56, 57] and reinforce the validity of the findings obtained from Bayesian network analysis.

This study has some limitations. First, the findings cannot be generalized because the study population was limited to individuals from specific regions. Second, although confounders were determined according to four criteria to minimize the risk of reverse causality, unanticipated biases cannot be eliminated because of the limitations of the causal diagram. Third, although we conducted Bayesian network analyses, it was impossible to conclude a causal association because of the study’s cross-sectional and observational design. Finally, although we defined periodontal disease as a probing depth ≥ 4 mm based on the clinical cutoff, this approach simplifies a condition with varying degrees of severity. Further studies analyzing pocket depth as a continuous variable are warranted.

## Conclusion

To the best of our knowledge, this is the first study to comprehensively analyze the association between antioxidants and periodontal diseases. We found an inverse association between the levels of blood carotenoids, especially lutein and lycopene, and prevalence of periodontal diseases. Similarly, skin carotenoid levels, which are measured noninvasively and easily, are inversely associated with the prevalence. Furthermore, Bayesian network analysis suggests that this association may be mediated by inflammation and oral dysbiosis. However, because this study population was limited to individuals from a specific region in Japan, further studies are needed to confirm the generalizability of the findings.

## Supplementary Material

Additional file 1: Additional file 1. Confounder selection. *Oral care habits referred to the following three variables, number of brushings per day, use of floss and interdental brush, and dental examinations in the last 1 year. Additional file 2. List of variables used in Bayesian networks analysis. Additional file 3. Prevalence of periodontal diseases according to age, sex, smoking status, and body mass index. PPD, probing pocket depth. Additional file 4. Adjusted odds ratios for periodontal diseases by blood antioxidants concentrations, skin carotenoid level, and vegetable intake. Additional file 5. Adjusted odds ratios for periodontal diseases by blood and skin carotenoid levels and dietary intakes. Additional file 6. Adjusted odds ratios of periodontal diseases based on subgroup. Only the odds ratios for the fifth quintile relative to the first quintile of blood carotenoid, skin carotenoid, blood lutein, and blood lycopene levels are shown. Age, sex, smoking and drinking status, sugar intake, educational background, and oral care habits were considered as confounders. Dots and whiskers indicate adjusted OR and 95% confidence intervals (CI), respectively. The interaction p indicates the statistical significance of the interaction term derived from the multivariate logistic regression model. OR, odds ratio. Additional file 7. Result of Bayesian network analysis. Additional file 8. Boxplots of salivary immunoglobulin A based on quintile of blood lutein and lycopene levels.

## Data Availability

All data in this study are available upon request from the Hirosaki University COI Program Institutional Data Access/Ethics Committee (contact via e-mail: coi@hirosaki-u.ac.jp) for researchers who meet the criteria for access to the data. The data cannot be shared publicly because of ethical concerns.

## Abbreviations

BMI: Body mass index
CI: Confidence interval
IHPP: Iwaki Health Promotion Project
OR: Odds ratio
FFQ: Food Frequency Questionnaire
