# Supplementary material for: Blood and skin carotenoid levels are inversely associated with the prevalence of periodontal diseases in populations with normal occlusion: a cross-sectional analysis from the Iwaki health promotion project
Source: Nutr J. 2026 Jan 28;25:26. doi: 10.1186/s12937-026-01285-y (PMC12924582; doi:10.1186/s12937-026-01285-y)
Supplement: Supplementary file 1 — Additional file 1: Additional file 1. Confounder selection. *Oral care habits referred to the following three variables, number of brushings per day, use of floss and interdental brush, and dental examinations in the last 1 year. Additional file 2. List of variables used in Bayesian networks analysis. Additional file 3. Prevalence of periodontal diseases according to age, sex, smoking status, and body mass index. PPD, probing pocket depth. Additional file 4. Adjusted odds ratios for periodontal diseases by blood antioxidants concentrations, skin carotenoid level, and vegetable intake. Additional file 5. Adjusted odds ratios for periodontal diseases by blood and skin carotenoid levels and dietary intakes. Additional file 6. Adjusted odds ratios of periodontal diseases based on subgroup. Only the odds ratios for the fifth quintile relative to the first quintile of blood carotenoid, skin carotenoid, blood lutein, and blood lycopene levels are shown. Age, sex, smoking and drinking status, sugar intake, educational background, and oral care habits were considered as confounders. Dots and whiskers indicate adjusted OR and 95% confidence intervals (CI), respectively. The interaction p indicates the statistical significance of the interaction term derived from the multivariate logistic regression model. OR, odds ratio. Additional file 7. Result of Bayesian network analysis. Additional file 8. Boxplots of salivary immunoglobulin A based on quintile of blood lutein and lycopene levels. [file 12937_2026_1285_MOESM1_ESM.docx]

Additional files


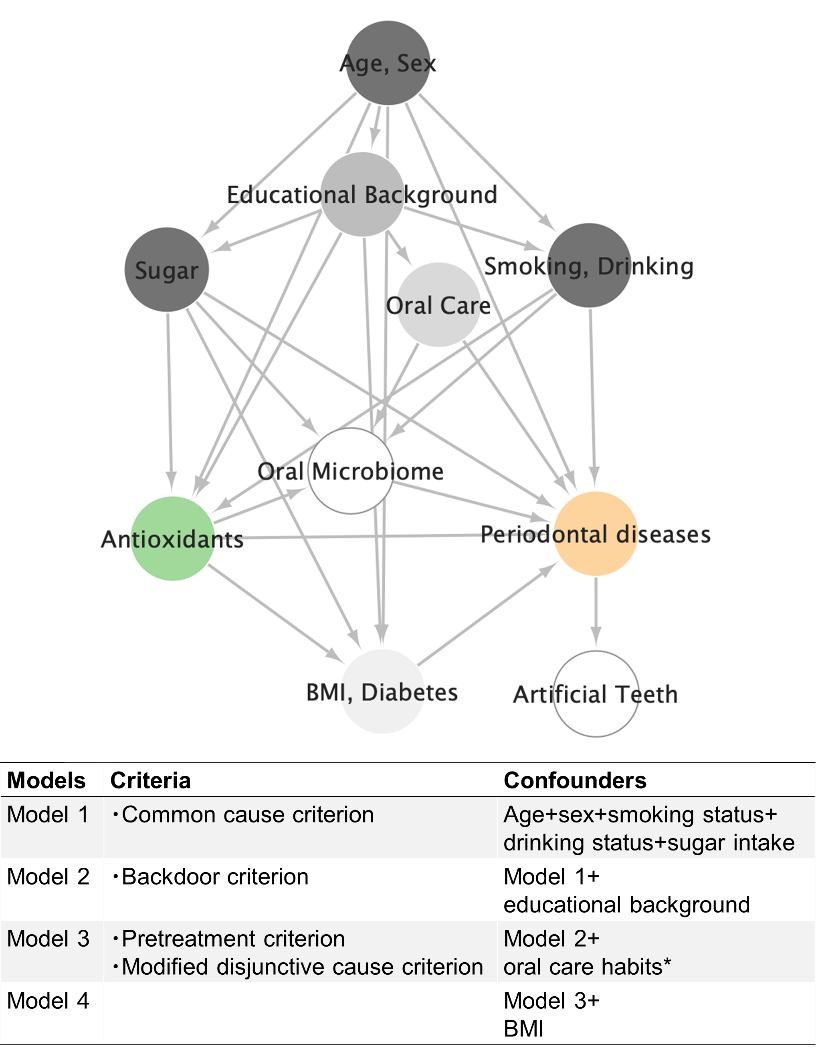


Additional file 1 Confounder selection

* Oral care habits referred to the following three variables, number of brushings per day, use of floss and interdental brush, and dental examinations in the last 1 year

Additional file 2 List of variables used in Bayesian networks analysis

| Category | Variables | Root nodes |
| --- | --- | --- |
| Demographic information | Age | ● |
|  | Sex | ● |
|  | Educational background | ● |
| Lifestyle factors | Smoking status |  |
|  | Drinking status |  |
|  | Body mass index |  |
| Dietary intakes | Grains |  |
|  | Potato |  |
|  | Sugar and sweeteners |  |
|  | Pulse |  |
|  | Nuts and bolts |  |
|  | Fruits |  |
|  | Mushrooms |  |
|  | Seaweed |  |
|  | Seafood |  |
|  | Meats |  |
|  | Eggs |  |
|  | Dairy |  |
|  | Oils and fats |  |
|  | Snacks |  |
|  | Alcohol |  |
|  | Tasty beverages |  |
|  | Seasoning and spices |  |
| Carotenoid | Blood carotenoid |  |
|  | Lutein |  |
|  | Lycopene |  |
|  | Skin carotenoid |  |
|  | Vegetable intakes |  |
| Oral care habits | Number of brushings |  |
|  | Use of floss and interdental brush | |
|  | Dental clinic (last one year) |  |
| Oral microbiome | *Neisseria* |  |
|  | *Prevotella* |  |
|  | *Streptococcus* |  |
|  | *Porphyromonas* |  |
|  | *Treponema* |  |
|  | *Tannerella* |  |
| Oral indices | Number of teeth |  |
|  | Eichner |  |
|  | Dental caries |  |
|  | Gingival bleeding |  |
|  | Calculus |  |
|  | Salivary IgA |  |
|  | Salivary flow rate |  |
|  | Oral Health Impact Profile 14 |  |
|  | Artificial tooth |  |
| Periodontal disease | Periodontal disease |  |


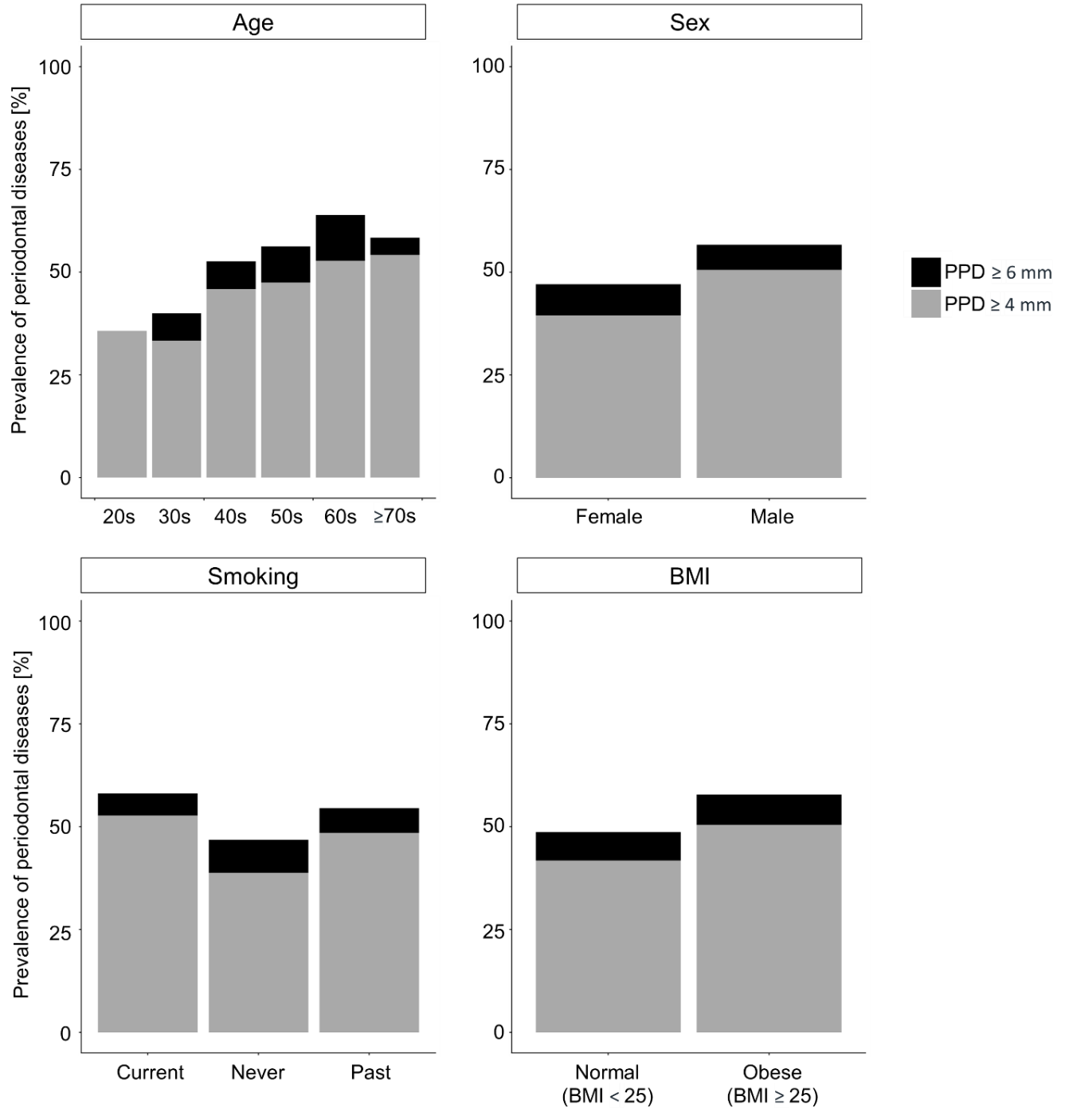


Additional file 3 Prevalence of periodontal diseases according to age, sex, smoking status, and body mass index.

PPD, probing pocket depth.

Additional file 4 Adjusted odds ratios for periodontal diseases by blood antioxidants concentrations, skin carotenoid level, and vegetable intake

|  |  | Model1 |  |  |  | Model2 |  |  |  | Model3 |  |  |  | Model4 |  |  |  |
| --- | --- | --- | --- | --- | --- | --- | --- | --- | --- | --- | --- | --- | --- | --- | --- | --- | --- |
|  | Quintile | Adjusted  OR | Lower | Upper | *p* | Adjusted  OR | Lower | Upper | *p* | Adjusted  OR | Lower | Upper | *p* | Adjusted  OR | Lower | Upper | *p* |
| Blood  carotenoid | 1st | Ref |  |  |  | Ref |  |  |  | Ref |  |  |  | Ref |  |  |  |
|  | 2nd | 0.86 | 0.47 | 1.58 | 0.631 | 0.89 | 0.48 | 1.65 | 0.717 | 0.83 | 0.44 | 1.55 | 0.555 | 0.80 | 0.43 | 1.51 | 0.496 |
|  | 3rd | 0.74 | 0.40 | 1.37 | 0.341 | 0.76 | 0.41 | 1.39 | 0.372 | 0.73 | 0.39 | 1.35 | 0.316 | 0.74 | 0.40 | 1.36 | 0.329 |
|  | 4th | 0.96 | 0.51 | 1.82 | 0.901 | 1.01 | 0.53 | 1.95 | 0.969 | 0.98 | 0.51 | 1.90 | 0.960 | 1.01 | 0.52 | 1.95 | 0.976 |
|  | 5th | 0.33 | 0.17 | 0.64 | 0.001 | 0.34 | 0.17 | 0.68 | 0.002 | 0.32 | 0.16 | 0.64 | 0.001 | 0.34 | 0.17 | 0.69 | 0.003 |
| Skin  carotenoid | 1st | Ref |  |  |  | Ref |  |  |  | Ref |  |  |  | Ref |  |  |  |
|  | 2nd | 0.65 | 0.35 | 1.19 | 0.159 | 0.65 | 0.36 | 1.20 | 0.169 | 0.64 | 0.35 | 1.18 | 0.157 | 0.63 | 0.34 | 1.16 | 0.137 |
|  | 3rd | 0.72 | 0.39 | 1.34 | 0.304 | 0.74 | 0.40 | 1.37 | 0.342 | 0.73 | 0.39 | 1.35 | 0.312 | 0.73 | 0.39 | 1.36 | 0.324 |
|  | 4th | 0.85 | 0.45 | 1.59 | 0.614 | 0.87 | 0.46 | 1.64 | 0.672 | 0.84 | 0.45 | 1.59 | 0.597 | 0.85 | 0.45 | 1.61 | 0.624 |
|  | 5th | 0.46 | 0.24 | 0.88 | 0.019 | 0.47 | 0.24 | 0.91 | 0.026 | 0.45 | 0.23 | 0.88 | 0.019 | 0.48 | 0.25 | 0.95 | 0.035 |
| Blood  vitamin A | 1st | Ref |  |  |  | Ref |  |  |  | Ref |  |  |  | Ref |  |  |  |
|  | 2nd | 1.11 | 0.60 | 2.05 | 0.735 | 1.12 | 0.61 | 2.07 | 0.716 | 1.12 | 0.60 | 2.06 | 0.726 | 1.09 | 0.59 | 2.03 | 0.776 |
|  | 3rd | 0.78 | 0.41 | 1.46 | 0.434 | 0.78 | 0.42 | 1.48 | 0.450 | 0.80 | 0.42 | 1.51 | 0.496 | 0.75 | 0.40 | 1.43 | 0.390 |
|  | 4th | 0.87 | 0.45 | 1.68 | 0.678 | 0.88 | 0.45 | 1.70 | 0.693 | 0.88 | 0.45 | 1.71 | 0.707 | 0.81 | 0.42 | 1.59 | 0.549 |
|  | 5th | 1.07 | 0.51 | 2.25 | 0.856 | 1.07 | 0.51 | 2.25 | 0.857 | 1.08 | 0.51 | 2.27 | 0.841 | 0.99 | 0.47 | 2.10 | 0.983 |
| Blood  vitamin C | 1st | Ref |  |  |  | Ref |  |  |  | Ref |  |  |  | Ref |  |  |  |
|  | 2nd | 1.54 | 0.84 | 2.83 | 0.164 | 1.58 | 0.86 | 2.92 | 0.142 | 1.49 | 0.80 | 2.77 | 0.204 | 1.46 | 0.78 | 2.71 | 0.237 |
|  | 3rd | 1.05 | 0.57 | 1.95 | 0.865 | 1.08 | 0.58 | 2.00 | 0.814 | 1.02 | 0.55 | 1.90 | 0.955 | 1.01 | 0.54 | 1.89 | 0.978 |
|  | 4th | 1.69 | 0.91 | 3.16 | 0.096 | 1.74 | 0.93 | 3.24 | 0.083 | 1.68 | 0.90 | 3.14 | 0.104 | 1.78 | 0.94 | 3.34 | 0.075 |
|  | 5th | 1.39 | 0.74 | 2.59 | 0.303 | 1.41 | 0.76 | 2.64 | 0.277 | 1.38 | 0.73 | 2.58 | 0.318 | 1.47 | 0.78 | 2.77 | 0.236 |
| Blood  vitamin E | 1st | Ref |  |  |  | Ref |  |  |  | Ref |  |  |  | Ref |  |  |  |
|  | 2nd | 0.84 | 0.46 | 1.56 | 0.587 | 0.87 | 0.47 | 1.61 | 0.662 | 0.87 | 0.46 | 1.62 | 0.660 | 0.86 | 0.46 | 1.62 | 0.650 |
|  | 3rd | 0.65 | 0.35 | 1.21 | 0.176 | 0.67 | 0.36 | 1.24 | 0.202 | 0.65 | 0.35 | 1.21 | 0.175 | 0.66 | 0.36 | 1.24 | 0.201 |
|  | 4th | 0.76 | 0.40 | 1.44 | 0.398 | 0.77 | 0.40 | 1.46 | 0.420 | 0.76 | 0.40 | 1.45 | 0.397 | 0.74 | 0.38 | 1.42 | 0.362 |
|  | 5th | 0.73 | 0.37 | 1.43 | 0.354 | 0.73 | 0.37 | 1.43 | 0.360 | 0.74 | 0.38 | 1.45 | 0.383 | 0.73 | 0.37 | 1.43 | 0.356 |
| Vegetable  intake | 1st | Ref |  |  |  | Ref |  |  |  | Ref |  |  |  | Ref |  |  |  |
|  | 2nd | 0.80 | 0.44 | 1.47 | 0.481 | 0.82 | 0.45 | 1.50 | 0.513 | 0.78 | 0.42 | 1.44 | 0.431 | 0.81 | 0.44 | 1.49 | 0.494 |
|  | 3rd | 1.20 | 0.65 | 2.22 | 0.565 | 1.23 | 0.66 | 2.28 | 0.517 | 1.23 | 0.66 | 2.29 | 0.514 | 1.22 | 0.66 | 2.28 | 0.526 |
|  | 4th | 1.28 | 0.69 | 2.40 | 0.436 | 1.32 | 0.70 | 2.49 | 0.385 | 1.30 | 0.68 | 2.45 | 0.426 | 1.29 | 0.68 | 2.45 | 0.430 |
|  | 5th | 0.64 | 0.33 | 1.21 | 0.168 | 0.66 | 0.34 | 1.26 | 0.206 | 0.63 | 0.33 | 1.20 | 0.160 | 0.62 | 0.32 | 1.20 | 0.157 |

Red text indicates *p* < 0.05.

Additional file 5 Adjusted odds ratios for periodontal diseases by blood and skin carotenoid levels and dietary intakes

|  |  | Model 1 |  |  |  | Model 2 |  |  |  | Model 3 |  |  |  | Model 4 |  |  |  |
| --- | --- | --- | --- | --- | --- | --- | --- | --- | --- | --- | --- | --- | --- | --- | --- | --- | --- |
| Category | Variables | Adjusted OR | Lower | Upper | *p* | Adjusted OR | Lower | Upper | *p* | Adjusted OR | Lower | Upper | *p* | Adjusted OR | Lower | Upper | *p* |
| Serum | Carotenoid (Total) | 0.33 | 0.17 | 0.64 | 0.001 | 0.34 | 0.17 | 0.68 | 0.002 | 0.32 | 0.16 | 0.64 | 0.001 | 0.34 | 0.17 | 0.69 | 0.003 |
|  | Lutein | 0.48 | 0.25 | 0.92 | 0.027 | 0.49 | 0.26 | 0.93 | 0.030 | 0.50 | 0.26 | 0.97 | 0.040 | 0.56 | 0.29 | 1.11 | 0.095 |
|  | Zeaxanthin | 0.76 | 0.42 | 1.40 | 0.386 | 0.77 | 0.42 | 1.42 | 0.409 | 0.78 | 0.43 | 1.44 | 0.427 | 0.82 | 0.45 | 1.52 | 0.537 |
|  | Beta Cryptoxanthin | 0.79 | 0.41 | 1.53 | 0.489 | 0.82 | 0.42 | 1.58 | 0.549 | 0.80 | 0.41 | 1.56 | 0.511 | 0.86 | 0.44 | 1.69 | 0.667 |
|  | Alpha Carotene | 0.64 | 0.34 | 1.20 | 0.167 | 0.66 | 0.35 | 1.25 | 0.203 | 0.63 | 0.33 | 1.18 | 0.150 | 0.67 | 0.35 | 1.29 | 0.232 |
|  | Beta Carotene | 0.54 | 0.26 | 1.10 | 0.088 | 0.56 | 0.27 | 1.15 | 0.114 | 0.54 | 0.26 | 1.11 | 0.093 | 0.60 | 0.29 | 1.27 | 0.181 |
|  | Lycopene | 0.47 | 0.25 | 0.86 | 0.015 | 0.48 | 0.26 | 0.89 | 0.020 | 0.49 | 0.26 | 0.91 | 0.024 | 0.51 | 0.27 | 0.95 | 0.033 |
| Skin | Carotenoid | 0.46 | 0.24 | 0.88 | 0.019 | 0.47 | 0.24 | 0.91 | 0.026 | 0.45 | 0.23 | 0.88 | 0.019 | 0.48 | 0.25 | 0.95 | 0.035 |
| Questionnaire | Vegetable | 0.64 | 0.33 | 1.21 | 0.168 | 0.66 | 0.34 | 1.26 | 0.206 | 0.63 | 0.33 | 1.20 | 0.160 | 0.62 | 0.32 | 1.20 | 0.157 |
|  | Green and yellow vegetables | 1.16 | 0.63 | 2.17 | 0.632 | 1.20 | 0.64 | 2.24 | 0.571 | 1.16 | 0.62 | 2.18 | 0.637 | 1.17 | 0.63 | 2.20 | 0.618 |
|  | Tomato | 0.65 | 0.34 | 1.25 | 0.197 | 0.67 | 0.35 | 1.30 | 0.239 | 0.67 | 0.34 | 1.29 | 0.231 | 0.69 | 0.35 | 1.34 | 0.269 |
|  | Others | 0.72 | 0.37 | 1.39 | 0.322 | 0.76 | 0.39 | 1.48 | 0.412 | 0.75 | 0.38 | 1.47 | 0.400 | 0.76 | 0.39 | 1.49 | 0.424 |
|  | Lycopene | 1.02 | 0.56 | 1.86 | 0.955 | 1.02 | 0.56 | 1.87 | 0.943 | 1.01 | 0.55 | 1.86 | 0.967 | 1.02 | 0.55 | 1.87 | 0.959 |
|  | Dietary Fiber | 0.53 | 0.27 | 1.06 | 0.073 | 0.55 | 0.27 | 1.10 | 0.090 | 0.53 | 0.26 | 1.05 | 0.070 | 0.54 | 0.27 | 1.08 | 0.079 |

Only the odds ratios for the fifth quintile relative to the first quintile of blood and skin carotenoid and vegetables intake are shown. The results of blood carotenoid (total), skin carotenoid, and vegetable intake were restatements of the results shown in Fig. 2 and Additional files 4. Red text indicates *p* < 0.05.


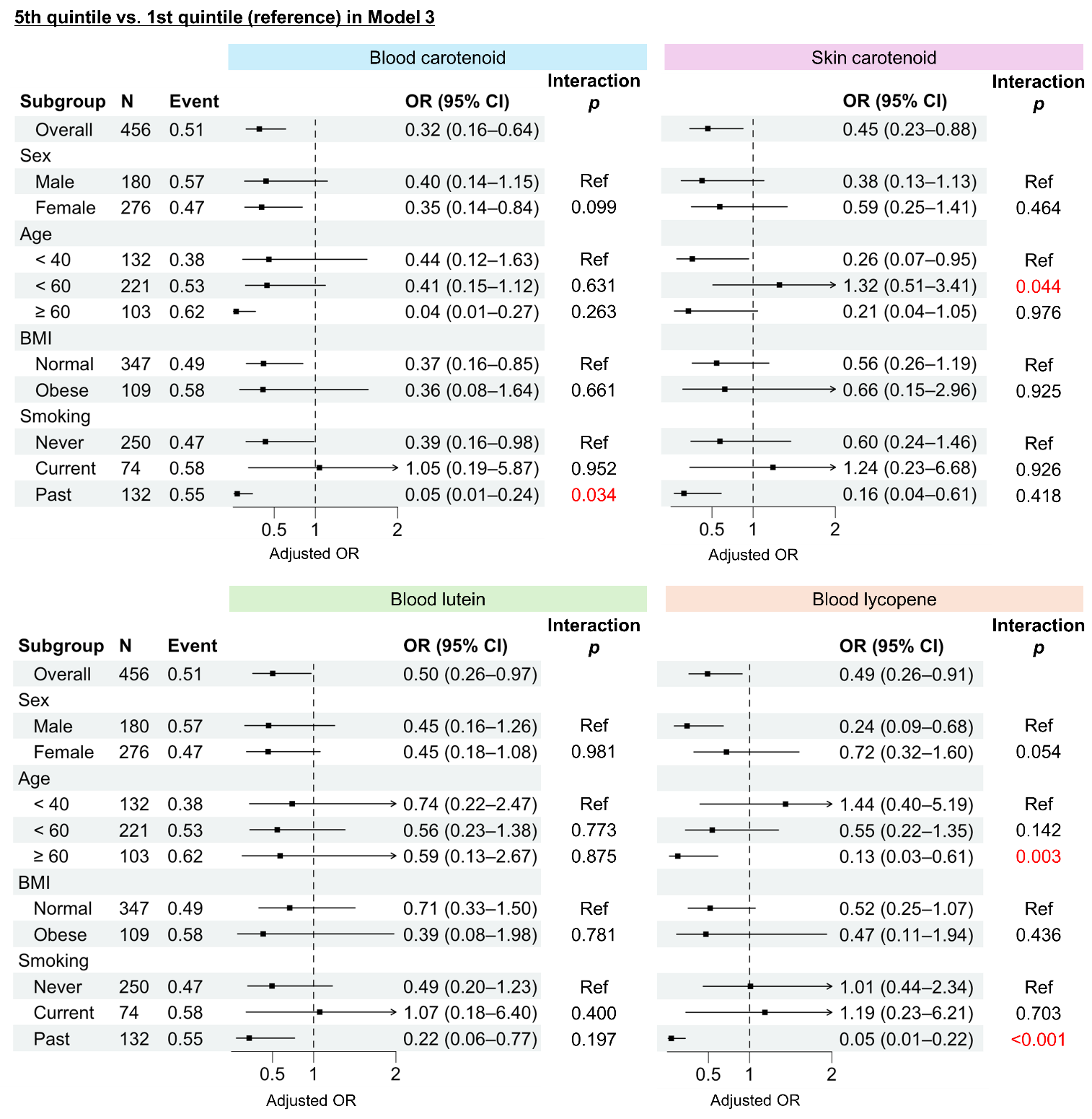


Additional file 6 Adjusted odds ratios of periodontal diseases based on subgroup

Only the odds ratios for the fifth quintile relative to the first quintile of blood carotenoid, skin carotenoid, blood lutein, and blood lycopene levels are shown. Age, sex, smoking and drinking status, sugar intake, educational background, and oral care habits were considered as confounders. Dots and whiskers indicate adjusted OR and 95% confidence intervals (CI), respectively. The interaction *p* indicates the statistical significance of the interaction term derived from the multivariate logistic regression model. OR, odds ratio.


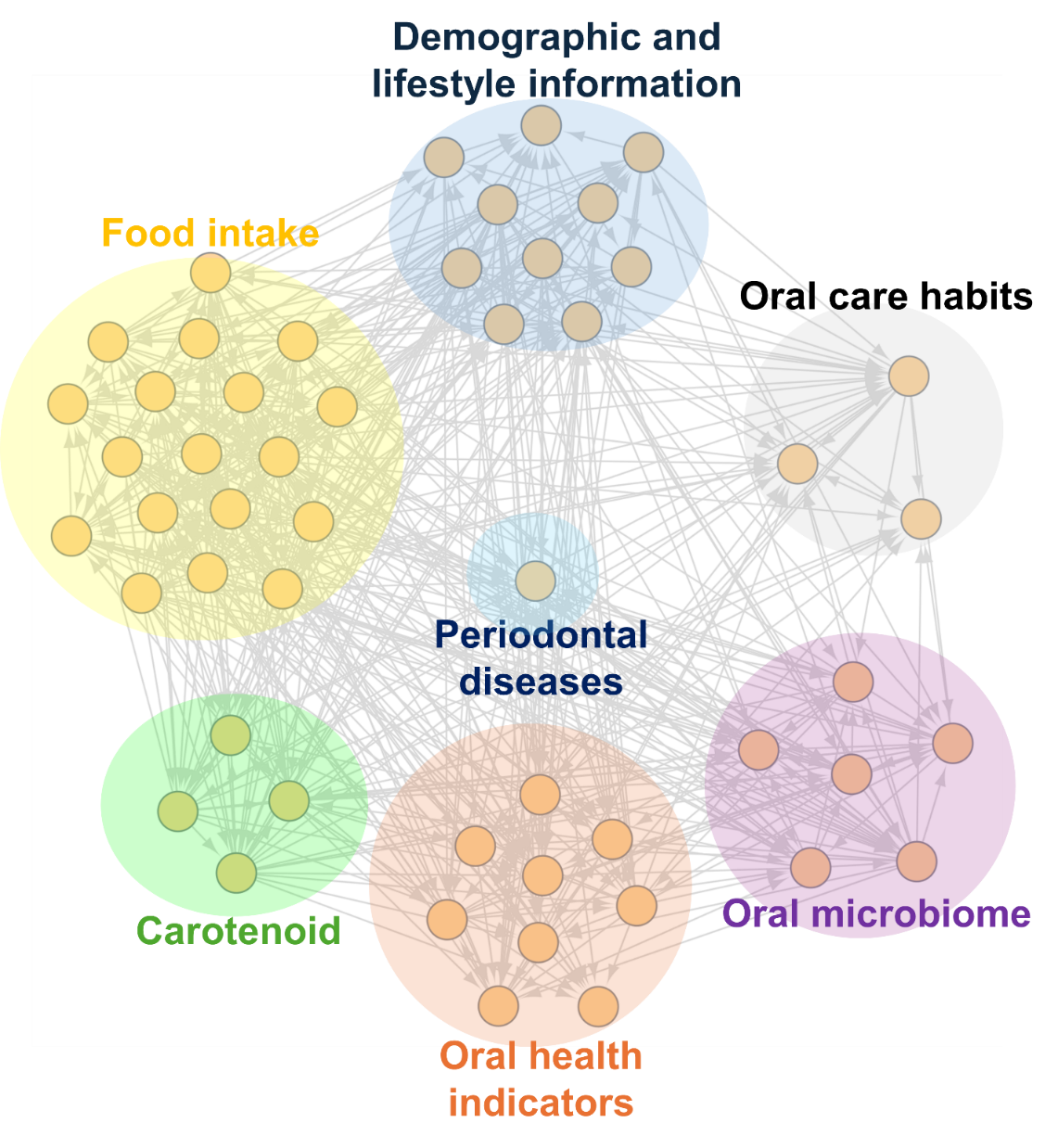


Additional file 7 Result of Bayesian network analysis


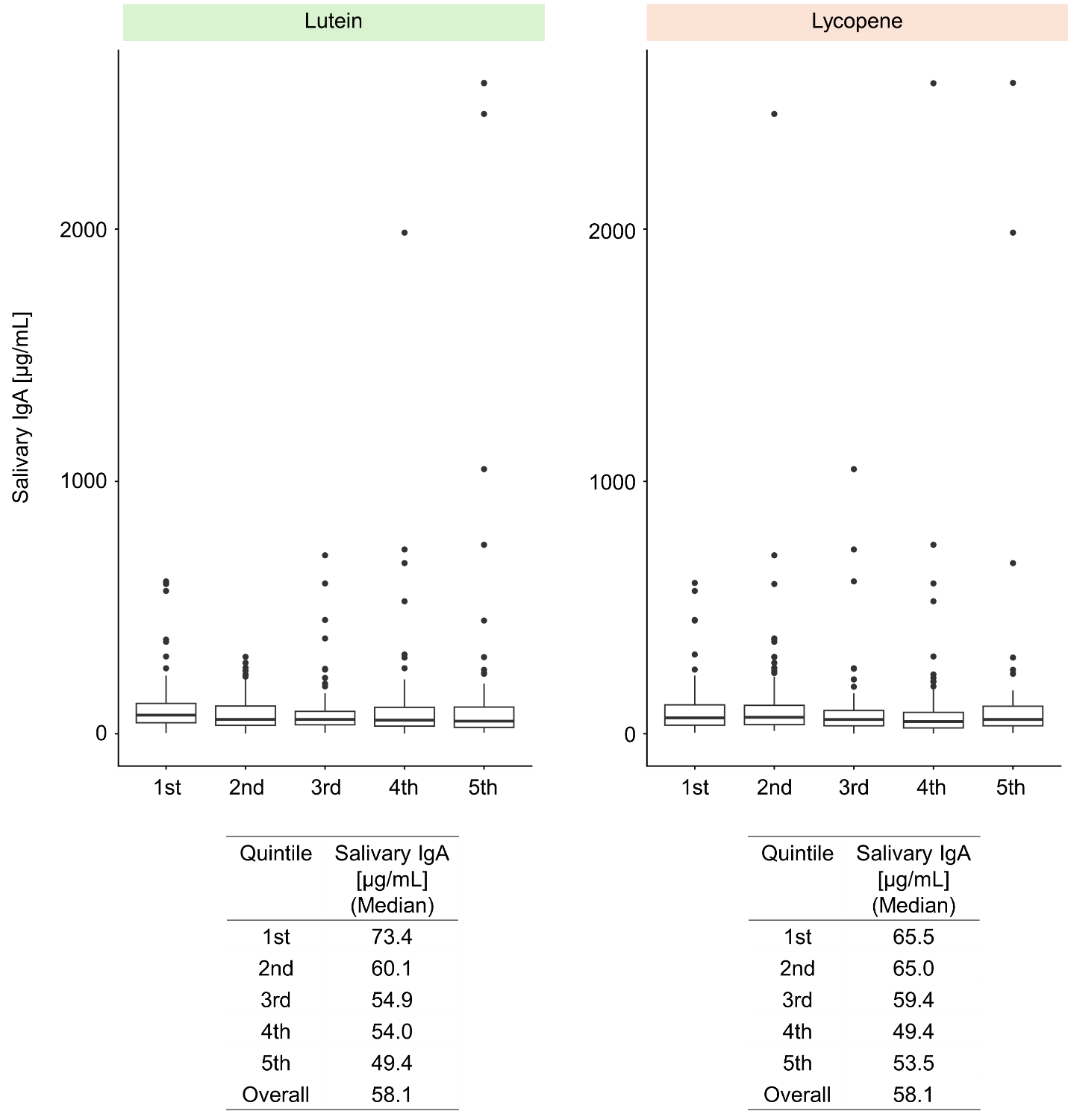


Additional file 8 Boxplots of salivary immunoglobulin A based on quintile of blood lutein and lycopene levels
